# A method for successfully implanting an implantable cardioverter-defibrillator wrapped with an expanded polytetrafluoroethylene sheet in a patient with metal allergy

**DOI:** 10.1016/j.jccase.2023.04.001

**Published:** 2023-04-29

**Authors:** Kei Morishita, Akiko Ishihara, Takatoshi Unno, Takahiko Murakami, Kensuke Okada, Hiroshi Matsunaga, Kazuo Asada, Yasutoshi Omori, Yoshiro Kamoi, Takahiro Tanaka

**Affiliations:** Department of Cardiology, Showa General Hospital, Tokyo, Japan

**Keywords:** Contact dermatitis, Cardiac implantable electronic device, Shock impedance, Congenital long QT syndrome, Torsades de pointes

## Abstract

Contact dermatitis is a severe complication of cardiac-device implantation that may be observed in patients with metal allergies. Some studies have suggested that wrapping cardiac devices with expanded polytetrafluoroethylene (ePTFE) sheets is effective in preventing contact dermatitis. Most of these studies involved pacemakers, whereas those on implantable cardioverter-defibrillators (ICDs) are rare. Herein, we report a method for the successful implantation of an ICD wrapped with an ePTFE sheet in a patient with metal allergy. The metal part of the ICD generator was tightly wrapped with an ePTFE sheet, which was sewn with ePTFE sutures approximating the edges of the generator. After the wrapping procedure, the patient entered the operating room, and the generator and an ePTFE-coated dual-coil shock lead were implanted via a standard procedure. The shock impedance in the coil-to-can vector was high immediately after the implantation, but it reduced to less than half of its initial value over a period of two weeks post-surgery. The patient did not develop any new skin problems during the 20-month follow-up. This is a method for successfully preventing contact dermatitis; however, attention to the associated high risk of infection is required.

**Learning objective:**

Wrapping an implantable cardioverter-defibrillator with an expanded polytetrafluoroethylene sheet was effective in preventing contact dermatitis after implantation. The shock impedance in the coil-to-can vector was high immediately after implantation but reduced to approximately half of its initial value with time.

## Introduction

Studies have reported skin ulcers or fluid accumulation in metal allergy patients due to contact dermatitis after cardiac-device implantation [1], [2], [3]. These patients often experience adverse outcomes, such as bacterial infection, exposure to generators, and sometimes death, making the extraction of generators and leads necessary [1], [4]. Several studies have suggested that wrapping cardiac devices with expanded polytetrafluoroethylene (ePTFE) sheets is effective in preventing contact dermatitis [5], [6]. Most of these studies have described the use of ePTFE with pacemakers (PMs), whereas studies on implantable cardioverter-defibrillators (ICDs) are rare. We report a successful method of implantation of an ICD wrapped with an ePTFE sheet in a patient with metal allergy. The report also provides an analysis of the time course of the shock impedance post-implantation.

## Case report

A 56-year-old female patient visited our hospital with a complaint of dyspnea. She was diagnosed with heart failure caused by permanent atrial fibrillation (AF) and severe mitral regurgitation (MR) after some outpatient examinations and decided to undergo cardiac surgery.

The patient had received medical therapy for heart failure for the preceding 10 years and underwent radiofrequency catheter ablation for atrial flutter 10 years prior, and plain balloon angioplasty of the right coronary artery #4PD for acute myocardial infarction 4 years prior. Her medications included bisoprolol, eplerenone, furosemide, pimobendan, and warfarin. She had a history of metal allergy, which developed intense erythema in areas of contact with metal ornaments and subsided on removal. She experienced an episode of anaphylactic shock after an intravenous injection of a contrast medium that required adrenaline administration. She also reported a history of alcohol allergy and heparin-induced-thrombocytopenia. She reported no previous episodes of syncope and no family history of sudden death or arrhythmia. On admission, her chest radiography showed cardiomegaly (cardiothoracic ratio: 60.1 %), and a 12‑lead electrocardiogram (ECG) revealed AF (QTc = 475 ms). Transthoracic echocardiography demonstrated severe MR due to P2–3 prolapse. Stress myocardial scintigram revealed no evidence of induced ischemia. Consequently, mitral valve replacement, tricuspid annuloplasty, and left atrial appendage ligation were scheduled.

The surgical procedures were successfully performed; however, on postoperative day 3, a 12‑lead ECG showed worsening of QT prolongation (QTc = 558 ms). The patient was initially asymptomatic but suddenly developed Torsades de pointes after R-on-T, progressed to ventricular fibrillation (VF), and lost consciousness.

VF was terminated by defibrillation, and the patient regained consciousness. Blood tests immediately after VF revealed normal levels of serum electrolytes. Magnesium (1-g bolus) was administered intravenously, followed by a continuous intravenous injection of lidocaine, amiodarone, and propofol; and overdrive pacing with a temporary pacemaker was provided.

ECGs were recorded repeatedly, and QTc duration remained longer than 500 ms. Genetic testing showed positive for LQT1. She was diagnosed with long QT syndrome, and an ICD implantation for secondary prevention was planned.

However, the patient's history of metal allergy raised concerns that the ICD generator and coils would develop contact dermatitis.

The patient had undergone an artificial valve replacement and was at a high risk of infective endocarditis after a bacterial infection associated with contact dermatitis.

The patient had adequate subcutaneous tissues that allowed the device to have a safe distance from the epidermis. Furthermore, we identified several risk factors for bleeding, including warfarinization and thrombocytopenia. Therefore, we created a pocket in the subcutaneous space, not in the subfascial space, to minimize the potential for structural trauma and accidental hemorrhage.

Although the S-ICD could have been chosen, the coils provide a disadvantage as the distance of the coils to the epidermis may potentially lead to allergic eipdermitis. Moreover, we needed ventricular pacing to suppress ventricular tachycardia. Therefore, the TV-ICD was chosen instead. To prevent contact dermatitis, we decided to wrap the generator (RESONATE™ EL ICD VR, Boston Scientific, Marlborough, MA, USA) with an ePTFE sheet (GORE® PRECLUDE® Pericardial Membrane 15.0 cm × 20.0 cm × 0.1 mm, W. L. Gore & Associates, Flagstaff, AZ, USA) and use an ePTFE-coated dual-coil shock lead (RELIANCE 4-FRONT™G, Boston Scientific).

**Fig. 1:**
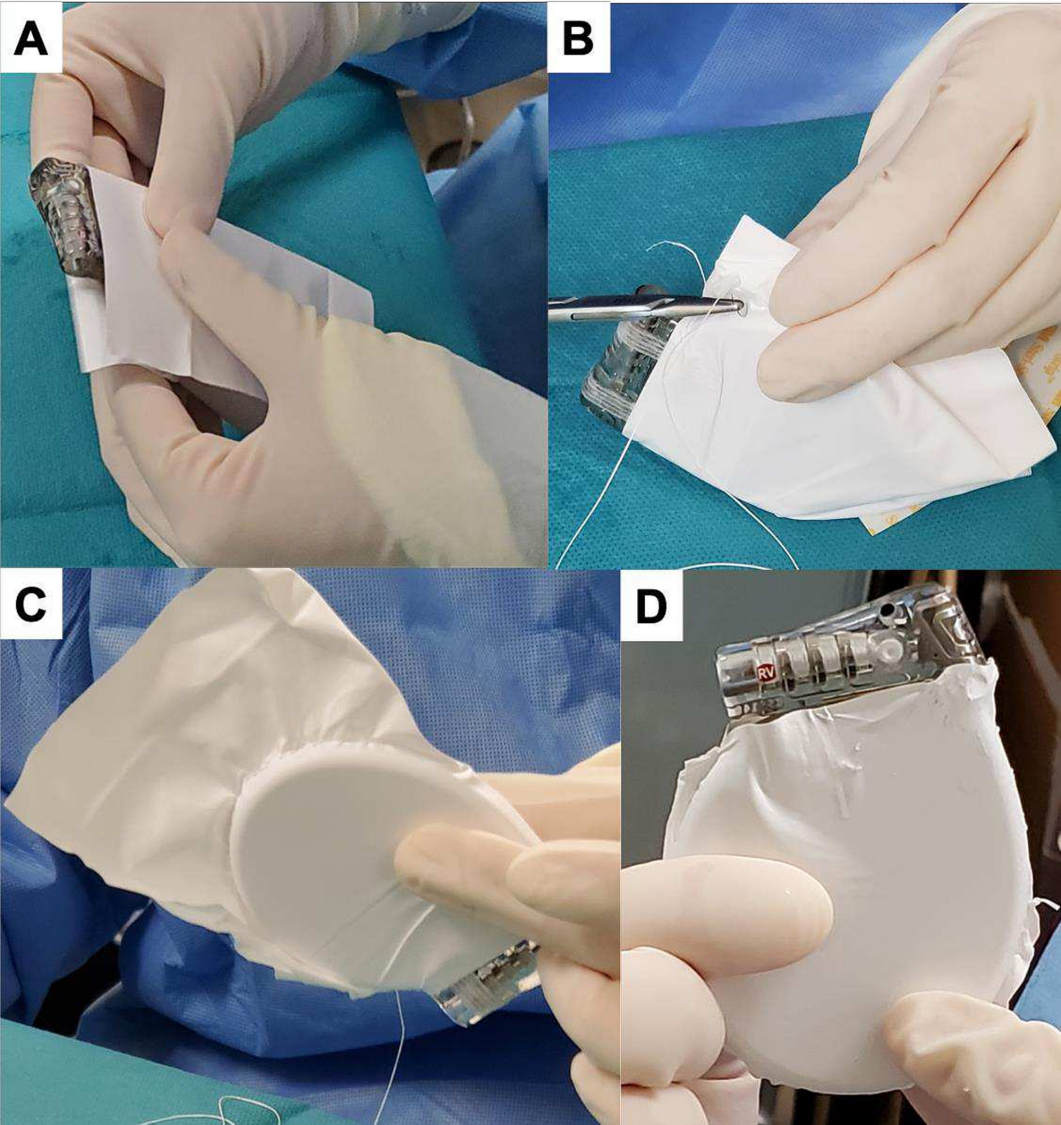
Operating room before the patient entered. The generator of the implantable cardioverter-defibrillator was wrapped with the expanded polytetrafluoroethylene sheet and sewn up tightly.

On the day of implantation, prior to the patient entering the operating room, the wrapping procedure was performed in a clean operative field. The metal part of the ICD generator was tightly wrapped inside an ePTFE sheet folded in half (Fig. 1A), and both sides of the sheet were sewn with ePTFE sutures (GORE-TEX® Suture 5M12, W. L. Gore & Associates) close to the edges of the generator (Fig. 1B, C) by half-back stitching every 5 mm, to reduce the amount of ePTFE used. The margins of the sheet were cut off along the edges of the generator (Fig. 1D).

**Fig. 2:**
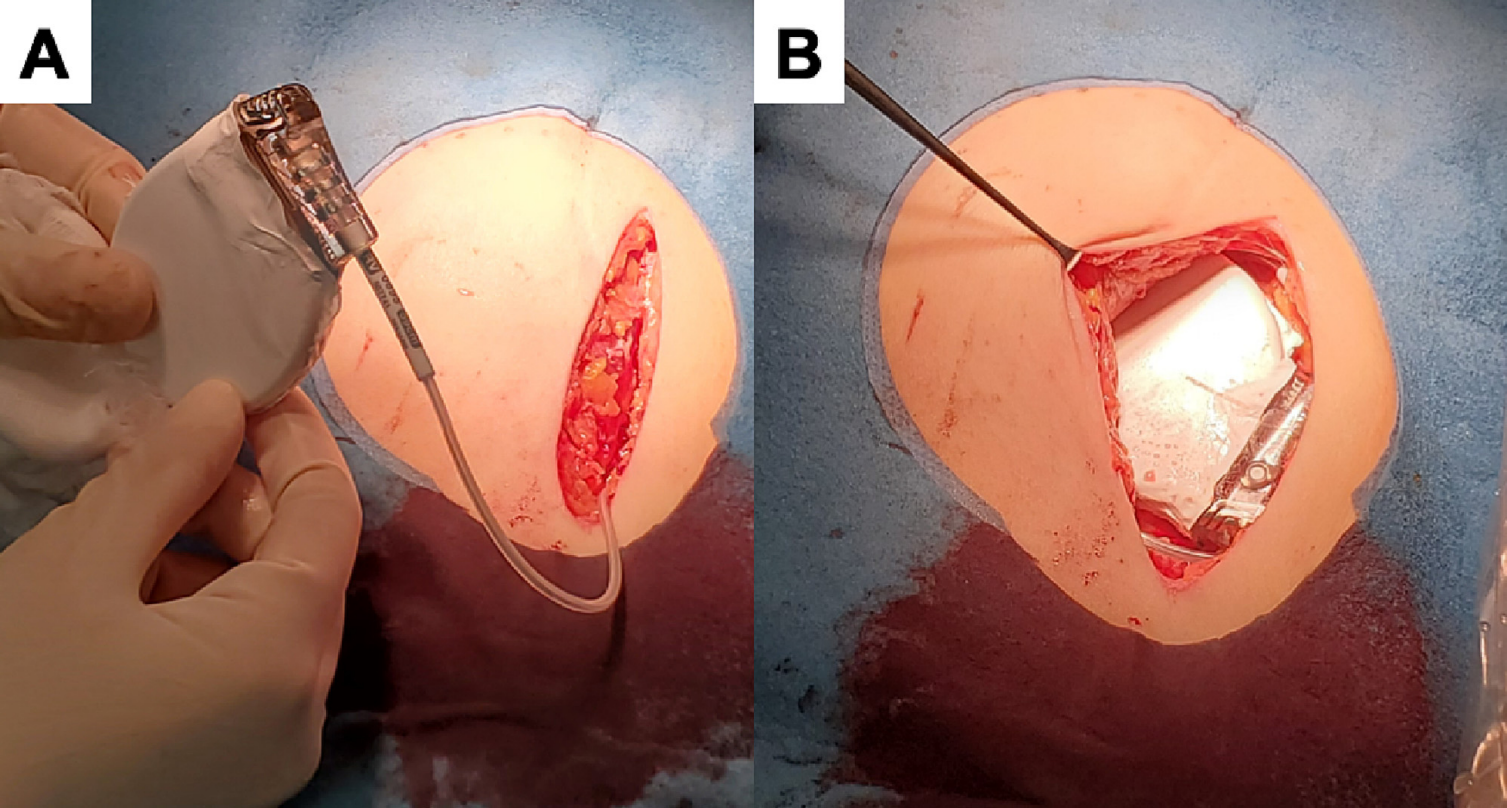
The insertion of implantable cardioverter-defibrillator wrapped with an expanded polytetrafluoroethylene sheet into the pocket.

After the patient entered the operating room, a subcutaneous pocket was created, and the shock lead was inserted transvenously into the right ventricular apex as per standard procedures. The generator wrapped with the ePTFE sheet was then connected to the lead (Fig. 2A) in the subcutaneous pocket (Fig. 2B) and fixed to the pectoral fascia. The surgical wound was closed with triple-layered closure.

As shown in Table 1, the coil-to-can shock impedance was high immediately after implantation, and we postponed discharge to observe the change in the impedance. After two weeks, the shock impedance reduced significantly to less than half of its initial value.

**Table 1:** Shock impedance.

	Shock impedance (Ω)			
	Day of operation	After 2 weeks	After 8 months	After 14 months
RV coil → RA coil	41	54	60	63
RV coil → can	174	83	83	85
RA coil → can	166	74	78	80

A defibrillation test was performed in the coil-to-coil vector, and VF was successfully terminated with a single 17-J shock.

The postoperative period was uneventful, with no skin problems, and the patient was discharged 22 days after the ICD operation (total duration of hospital stay: 28 days). The patient had no skin problems during the 20-month follow-up.

## Discussion

Cardiac-device-associated contact dermatitis is a delayed hypersensitivity reaction caused by the components of the implanted devices [7], with an estimated prevalence of 1.5–2.5 % [4]. Medications such as steroids or histamine receptor inhibitors are not recommended for long-term use, and, in principle, the generator and leads must be removed [1], [8].

In a retrospective study by Yashiro et al. [9], ePTFE-wrapped devices were used in 19 of the 4497 patients who underwent cardiac-device implantation. Among them, 14 patients had PMs, and 2 had ICD implants.

No allergic or immune reactions were observed during a mean follow-up of 46 months. However, device infections developed in three patients (15 %) at a rate significantly higher than the normal cardiac-device implantation (1–1.3 %) [10]. The authors speculate that the increased infection risk may be attributed to two factors: the additional process in the operative procedure and the environment, such as exudate pools and irregular surfaces of devices that favor microbial adherence.

To our knowledge, this is the first case report that describes the detailed method of ePTFE-wrapped ICD implantation.

Referring to a previous study [9], we applied the wrapping procedure before the patient entered the operating room to reduce the risk of infection by shortening the duration of surgery. Further, the sheet was tightly sealed to prevent creating a dead space. These efforts might have contributed to the favorable postoperative outcome.

Unique to ICDs, one concern on ePTFE-wrapping is that it increases the shock impedance in the coil-to-can vector and interferes with the shock efficacy. We chose a dual-coil lead and set the optimal shock vector, which was from the right ventricular coil to the superior vena cava coil (coil-to-coil). The coil-to-can shock impedance was high immediately after implantation but reduced to less than half of its initial value with time (Table 1). The ePTFE sheets have open microstructure pores large enough to accommodate cell growth on the patch surface. This enables the patch to become integrated with native tissue. Thus, the shock impedance and defibrillation threshold of the ePTFE-coated ICD should be almost equal to that of the normal cases. However, the time course was unknown. In our case, the shock impedance reduced significantly after two weeks.

To the best of our knowledge, no other studies have addressed the effect of ePTFE-wrapping on shock impedance. Our case suggests that shock impedance increase can be prevented by performing the correct wrapping technique.

In the aforementioned study [9], 11 of the 19 cases of ePTFE-wrapped device implantation were performed in patients who required device revision due to contact dermatitis after previous implantation (secondary prevention), while 7 cases were initial implantation in patients with history of metal allergies.

In cases of secondary prevention, the need for ePTFE-wrapping is high; thus, the decision to perform ePTFE-wrapping is simple. However, in cases of initial implantation, the decision to perform ePTFE-wrapping is difficult. Careful consideration should be given to each patient while assessing the risk of developing infection versus contact dermatitis.

## Conclusion

We report the case of a successful implantation of ePTFE-wrapped ICD in a patient with metal allergy. The patient developed no dermatological issues during the 20-month follow-up. The shock impedance in the coil-to-can vector was high, immediately after implantation; however, it reduced to slightly less than half of the initial value with time. Nevertheless, the high risk of infection associated with these cases requires attention.

## Declaration of competing interest

The authors declare no conflicts of interest.

## References

[1] Dogan P., Inci S., Kuyumcu M.S., Kus O. (2016). Contact dermatitis after implantable cardiac defibrillator implantation for ventricular tachycardia. Intractable Rare Dis Res.

[2] Andrews I.D., Scheinman P. (2011). Systemic hypersensitivity reaction (without cutaneous manifestations) to an implantable cardioverter-defibrillator. Dermatitis.

[3] Peters M.S., Schroeter A.L., van Hale H.M., Broadbent J.C. (1984). Pacemaker contact sensitivity. Contact Dermatitis.

[4] Grosse Meininghaus D.G., Kruells-Muench J., Peltroche-Llacsahuanga H. (2020). First-in-man implantation of a gold-coated biventricular defibrillator: difficult differential diagnosis of metal hypersensitivity reaction vs chronic device infection. HeartRhythm Case Rep.

[5] Ishii K., Kodani E., Miyamoto S., Otsuka T., Hosone M., Ogata K., Sato W., Matsumoto S., Tadera T., Ibuki C., Kusama Y., Atarashi H. (2006). Pacemaker contact dermatitis: the effective use of a polytetrafluoroethylene sheet. Pacing Clin Electrophysiol.

[6] Tamenishi A., Usui A., Oshima H., Ueda Y. (2008). Entirely polytetrafluoroethylene coating for pacemaker system contact dermatitis. Interact Cardiovasc Thorac Surg.

[7] Raja Y., Desai P.V., Glennon P.E. (2008). Pacemaker-mediated dermatitis. Europace.

[8] Kang J., Simpson C.S., Campbell D., Borici-Mazi R., Redfearn D.P., Michael K.A., Abdollah H., Baranchuk A. (2013). Cardiac rhythm device contact dermatitis. Ann Noninvasive Electrocardiol.

[9] Yashiro B., Shoda M., Tomizawa Y., Manaka T., Hagiwara N. (2012). Long-term results of a cardiovascular implantable electronic device wrapped with an expanded polytetrafluoroethylene sheet. J Artif Organs.

[10] Polyzos K.A., Konstantelias A.A., Falagas M.E. (2015). Risk factors for cardiac implantable electronic device infection: a systematic review and meta-analysis. Europace.

